# A novel bi-directional heterogeneous network selection method for disease and microbial association prediction

**DOI:** 10.1186/s12859-022-04961-y

**Published:** 2022-11-14

**Authors:** Jian Guan, Zhao Gong Zhang, Yong Liu, Meng Wang

**Affiliations:** School of Computer Science and Technology, Heilongjiang University, Harbin, China

**Keywords:** Bi-directional heterogeneous network, causal selection model, Potential microorganism disease prediction, Random walk

## Abstract

Microorganisms in the human body have a great impact on human health. Therefore, mastering the potential relationship between microorganisms and diseases is helpful to understand the pathogenesis of diseases and is of great significance to the prevention, diagnosis, and treatment of diseases. In order to predict the potential microbial disease relationship, we propose a new computational model. Firstly, a bi-directional heterogeneous microbial disease network is constructed by integrating multiple similarities, including Gaussian kernel similarity, microbial function similarity, disease semantic similarity, and disease symptom similarity. Secondly, the neighbor information of the network is learned by random walk; Finally, the selection model is used for information aggregation, and the microbial disease node pair is analyzed. Our method is superior to the existing methods in leave-one-out cross-validation and five-fold cross-validation. Moreover, in case studies of different diseases, our method was proven to be effective.

## Introduction

The microbial community is composed of bacteria, fungi, protozoa, and eukaryotes. It has an important impact on human beings in the fields of food, agriculture, environmental governance, and human health [1]. Microorganisms living in different organs of the human body can directly affect human health by regulating human immune system, drug metabolism and pathogen prevention [2]. Therefore, finding more links between microorganisms and diseases can not only help us better under- stand the pathogenesis of diseases, but also promote doctors’ diagnosis of diseases. In recent years, many computational methods have been proposed to explore the potential correlation in biological information. The existing calculation methods applied to microbial disease association are mainly divided into three categories. The first is based on fractional functions, the second is based on network algorithms, and the third is based on machine learning.

The first method, the Katz method proposed by Chen et al., is based on scoring function utilizing similarity and known association to predict the potential association between microorganism and disease. For association prediction, this method uses the number and length of paths between nodes in heterogeneous networks to predict [3]. Additionally, Li et al. [4] used Gaussian interaction profle kernel (GIP) similarity to build a weighted heterogeneous network for association prediction.

The second type of prediction method is to use machine learning technology to predict microbial disease association [5, 6]. Wang et al. [7] proposed a semi supervised model based on Laplace regularized least square algorithm. Peng et al. [8] combined multiple weak classifiers to form a strong classifier for prediction.

Lastly, methods based on random walk and matrix decomposition have also been explored to reveal potential microbial disease associations. Several methods use ordinary random walk [9], double random walk of logic function transformation [10] and random walk based on hypergraph [11] to predict potential microbial disease association. Qu et al. [12] used matrix decomposition and label propagation to infer potential associations. In addition, [13] proposed a method based on similarity constraint matrix decomposition to predict potential microRNA and disease associations. Several other methods have been proposed to predict microbial and disease associations based on network consistency projection [14] and multi similarity fusion tag propagation [15]. However, these methods still have some limitations. Because the sparsity of data and the singleness of methods limit the dissemination of information; these methods are difficult to extract the deep-seated Association of microorganisms (or diseases) from the data.

With the development of artificial intelligence, the method based on deep learning has been widely used in various fields. Long et al. proposed a framework to complete prediction based on graph attention network and inductive matrix [16]. Lei et al. [17] used the combination of node2vec algorithm and rule-based reasoning to predict potential associations. Liu et al. [18] have combined non negative matrix decomposition, random walk and capsule neural network to predict the association between microorganisms and diseases. A method based on multi-component graph attention network (GATMDA) was proposed to predict the potential association between microorganisms and diseases [19]. However, many similarities of various microorganisms (diseases) have not been fully utilized. Moreover, most of the previous methods rely on the known microorganism disease association for similarity calculation, thus these methods cannot achieve prediction when involving new diseases (or new microorganisms) due to the lack of training data.

In this paper, we propose a method (BDHNS), to predict microbial disease association based on a bi-directional microbial disease heterogeneous network and selection model. The contribution of our approach is mainly reflected in the following three aspects. First, we constructed a bi-directional heterogeneous microbial disease network based on the similarities between microorganisms and diseases. Different similarities can more comprehensively reflect the relationship between microorganisms and diseases from different angles. Secondly, based on an enhanced bi-directional random walk, we learn the neighbor topology information of microorganism and disease nodes in bi-directional heterogeneous networks from the two directions of microorganism disease and disease microorganism. The multi-feature fusion of microorganism and disease nodes is helpful for the final prediction of microorganism disease association. In heterogeneous networks, if all the neighbor information of each node is aggregated, the information of different types of nodes may also be aggregated, which will cause information redundancy. Therefore, lastly, we propose a graph convolution-based selection model to selectively aggregate neighbor information of disease (microorganism) nodes. The improved prediction performance is demonstrated by comparison with the most advanced models, ablation experiments, and case studies based on the Loocv and Five-fold cross-validation.

**Fig. 1:**
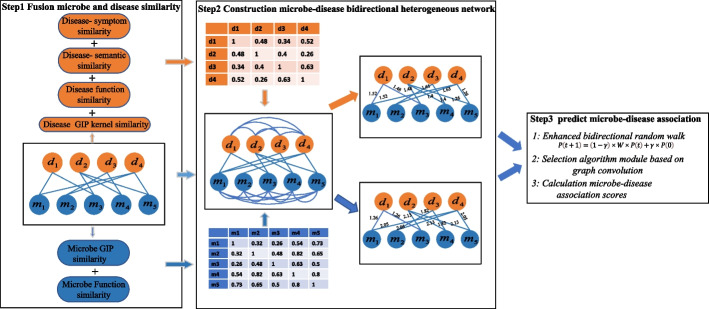
Overall flowchart of BDHNS. Step1: We fuse the calculated similarity of two microorganisms with the similarity of four diseases. Step2:We first build two one-way heterogeneous network respectively corresponding microorganisms and diseases, and then convert two one-way heterogeneous networks into a two-way heterogeneous network of microorganisms and diseases. Step3:We use the enhanced random walk and selection algorithm to predict the potential association between microorganisms and diseases

## Materials and methods

In order to predict the potential association between microorganisms and diseases, we propose a method based on a bi-directional random walk and selection model (Fig. 1). Firstly, a bi-directional heterogeneous network containing microorganisms and disease nodes is established based on multiple similarities to integrate the Gaussian kernel similarity of microorganisms, the functional similarity of microorganisms, the semantic similarity of diseases, and the similarity of disease symptoms. Secondly, we build an enhanced bi-directional random walk module to learn the neighbor topology information of microorganisms and disease nodes. Then, a graph convolution based selection model is proposed to selectively aggregate the neighbor topology and attribute information of each node in the network and calculate the association probability of node pairs.

### Microbial disease association data

We downloaded microbial disease association data from the database HMDAD, including 39 diseases and 292 microorganisms, covering 483 microbial disease associations. After removing duplicate records, we obtained 450 associations involving 39 diseases and 292 microorganisms. Subsequently, we used the data to construct the microbial disease association matrix *A*, if disease *d*(*i*) is associated with microorganism *m*(*j*), then $A(i,j)=1$, otherwise $A(i,j)=0$.

### Construction of bi-directional heterogeneous network

Based on the similarity of many microorganisms and diseases, we constructed a bi-directional heterogeneous microbial disease network $G=(V,E)$. Among them, node set *V* is composed of microbial node set ${V_m}$ and disease node set ${V_d}$. Edge ${e_{ij}} \in E$ represents the edge between a pair of nodes, ${V_i},{V_j} \in V$. Heterogeneous network *G* contains microbial-microbial similarity, disease-disease similarity and bi-directional association between microorganisms and diseases based on disease similarity and microbial similarity.

#### Microbial similarity

The more diseases are associated with two microorganisms, the more likely they are to demonstrate the same function. GIP similarity of microorganisms was calculated based on this assumption. The specific calculation process of GIP similarity matrix *GM* of microorganisms *m*(*i*) and *m*(*j*) is as follows:

1 $$GM(m(i,j)) = \exp \left( { - r_{m} \left\| {A(m(i)) - A(m(j))} \right\|^{2} } \right)$$

2 $$\begin{aligned}&{r_m} = {{{r_m}^\prime } / {\frac{1}{N}\sum \limits _{i = 1}^{{N_m}} {||A(m(i))|{|^2}} }} \end{aligned}$$

where *A*(*m*(*i*)) represents column *i* of *A*. ${r_m}^{'}$ is a parameter that affects ${r_m}$ normalization and ${r_m}^{'}=1$. ${N_m}$ indicates that the number of microorganisms is 292.

Information on the resident organs of microorganisms and their effects on disease can be obtained from HMDAD. If two microorganisms live in the same organ and are associated with similar diseases, there is a greater degree of similarity between them. In the same organ, if two microorganisms affect the same disease, the degree of similarity between them is 1, otherwise it is 0. The microbial function similarity matrix *FM* can be obtained by accumulating the effects of diseases on microorganisms in various organs. Then the FM is normalized to obtain the final *FM*. The normalization calculation process is as follows:

3 $$\begin{aligned} FM(m(i),m(j)) = \frac{{FM(m(i),m(j)) - \min (FM)}}{{\max (FM) - \min (FM)}} \end{aligned}$$

where ${\max (FM)}$ and ${\min (FM)}$ are the maximum and minimum values in the matrix *FM*.

Given microbial GIP similarity matrix *GM* and microbial functional similarity matrix *FM*, the final microbial similarity matrix *SM* is integrated as follows:

4 $$\begin{aligned} SM(i,j) = \left\{ \begin{array}{l} GM(i,j) ~ ~ ~ ~ ~ ~ ~ ~ ~ ~~ ~ ~ ~ ~~ ~ ~ ~ ~ ~ ~ ~ ~ ~if~ FM(i,j) = 0\\ (GM(i,j) + FM(i,j))/2~ ~ ~ ~ else \end{array} \right. \end{aligned}$$

#### Disease similarity calculation

Similar to the calculation method of microbial GIP similarity matrix, the calculation process is as follows:

5 $$GD(d(i,j)) = \exp \left( { - r_{d} \left\| {A(d(i)) - A(d(j))} \right\|^{2} } \right)$$

6 $$\begin{aligned}&{r_d} = {{{r_d}^\prime }/ {\frac{1}{N}\sum \limits _{i = 1}^{{N_d}} {||A(d(i))|{|^2}}}} \end{aligned}$$

where *A*(*d*(*i*)) represents row *i* of adjacency matrix *A*. ${{r_d}^{'} }$ set to 1. ${N_d}$ is 39, representing the number of diseases. Each disease can be represented by constructing a directed acyclic graph (*DAG*), which contains the disease and all its ancestral diseases [20]. Thus, we can calculate the semantic contribution of each disease in *DAG* to each disease in our data. The calculation process is as follows:

7 $$\begin{aligned} {D_D}(d) = \left\{ \begin{array}{l} 1~{{~~~~~~~~~~~~~~~~~~~~~~~~~~~~~~~~~~~~~~~~~~~~~~}}~if~d=D\\ \max \left\{ {\Delta \times {D_D}(d')|d' \in children~of~d} \right\} ~if~d \ne D \end{array} \right. {} \end{aligned}$$

where $\Delta$ is the semantic attenuation factor. The value of is set to 0.5.

By calculating the semantic contribution values of all diseases in *DAG*, the semantic values of diseases are calculated as follows:

8 $$\begin{aligned} {D_V}(D) = \sum \nolimits _{t \in {V_d}} {{D_D}(t)} \end{aligned}$$

where ${V_d}$ includes disease *D* and all its ancestral diseases. The more common semantics the two diseases *DAG* contains, the more similar the two diseases will be. The semantic similarity between the two diseases is calculated as follows:

9 $$\begin{aligned} DSS(d(i),d(j)) = \frac{{\sum \nolimits _{t \in T({d(i)}) \cap T({d(j)})} {{D_{(i)}}(t) + {D_{(j)}}(t)} }}{{{D_V}(D(i)) + {D_V}(D(j))}} \end{aligned}$$

Two similar diseases may interact with similar genes [21], and the disease function similarity matrix can be calculated based on the interaction between disease-related genes. Humannet v2.0 database contains gene interactions [22], in which each interaction has a related log likelihood score (LLS) to evaluate the probability of functional linkage between genes. We can get the relevant genomes of diseases *d*(*i*) and *d*(*j*) that ${G_i} = \{ {g_{i1}},{g_{i2}},...,{g_{im}}\}$ and ${G_j} = \{ {g_{j1}},{g_{j2}},...,{g_{jn}}\}$, respectively. where *m* is the number of genes in ${G_i}$ and *n* is the number of genes in ${G_j}$. The association between gene *g* and gene set $G = \{ {g_i},{g_2},...,{g_k}\}$ is as follows:

10 $$\begin{aligned} {F_G}(g) = \mathop {\max }\limits _{{g_i} \in G} (FSS((g,{g_i}))) \end{aligned}$$

where *FSS* represents the functional similarity score between genes, which is calculated as follows:

11 $$\begin{aligned} FSS({g_i},{g_j}) = \left\{ \begin{array}{l} 1~\mathrm{{ if i = j}}\\ LLS'({g_i},{g_j})\mathrm{{ ~if~i}} \ne \mathrm{{j}} \end{array} \right. \end{aligned}$$

where $LLS'$ is the standardization of gene *LLS*, which is calculated as follows:

12 $$\begin{aligned} LLS'({g_i},{g_j}) = \frac{{LLS({g_i},{g_j}) - LL{S_{\min }}}}{{LL{S_{\max }} - LL{S_{\min }}}} \end{aligned}$$

where $LL{S_{\max }}$ and $LL{S_{\min }}$ represent the maximum and minimum values in HumanNet respectively.

Finally, we calculate the disease functional similarity as follows:

13 $$\begin{aligned} DF(d(i),d(j)) = \frac{{\sum \limits _{{g_t} \in G(d(i))} {{F_{G({d(j)})}}({g_t}) + \sum \limits _{{g_t} \in G({d(j)})} {{F_{G(d( i ))}}({g_t})} } }}{{m + n}} \end{aligned}$$

By integrating disease GIP similarity, disease semantic similarity, disease symptom similarity (*TD*) and disease function similarity, the final disease similarity matrix *SD* is expressed as:

14 $$\begin{aligned} SD = \frac{{GD + DSS + TD + DF}}{4} \end{aligned}$$

where symptom-based diseases similarity TD was calculated using the associations of diseases and symptoms. The associations between diseases and symptoms is extracted from the human symptomatic disease network [23]. Therefore, we use the vectors of diseases related symptoms and refer to the method in [23] to calculate the similarity between disease-disease based on cosine similarity measurement, and then obtain disease symptom similarity (TD).

#### Bi-directional correlation calculation between microorganism and disease

For a given disease, the degree of correlation with different microorganisms is different. For example, for a given disease *d*(*i*), some microorganisms related to *d*(*i*) have a strong similarity relationship, while others have no or low similarity relationship with *d*(*i*). Therefore, the similarity topology between microorganism and disease is used to calculate the correlation between disease (microorganism) and microorganism (disease). Specifically, we constructed a bi-directional heterogeneous network containing disease and microbial nodes. When calculating the correlation degree of disease to microorganism, the edge weight transferred from the disease network $d(i)(i=1,2,...,{N_d})$ node to the microbial network $m(j)(j=1,2,...,{N_m})$ node is defined as the sum of the weights from the disease node associated with microorganism *m*(*j*) to the *d*(*i*) node. Similarly, we can also calculate the degree of association between microorganisms and disease direction. Therefore, two new adjacent matrices $A_{SD}^{'}$ and $A_{SM}^{'}$ can be obtained based on the similarity matrices *SD* and *SM*. The calculation process is as follows:

15 $$\begin{aligned}&A_{SD}^{'}(i,j) = \sum \limits _{k = 1}^{{n_d}} {SD(i,k){a_{kj}}} \end{aligned}$$

16 $$\begin{aligned}&A_{SM}^{'}(i,j) = \sum \limits _{k = 1}^{{n_m}} {{a_{ik}}SM(k,j)} \end{aligned}$$

where ${a_{ik}}$(${a_{jk}}$) is the element on row *i* and column *k* (row *k* and column *j*) of adjacent matrix *A*.

Given the bi-directional correlation matrix $A_{SD}^{'}$ and $A_{SM}^{'}$ of microorganisms and diseases,microbial similarity matrix *SM* and disease similarity matrix *SD*, a bi-directional heterogeneous microbial disease network can be established. The adjacency matrix of bi-directional heterogeneous networks is ${A_{all}}$,

17 $$\begin{aligned} {A_{all}} = \left( {\begin{array}{*{20}{c}} {SD}&{}{A_{SD}^{'}}\\ {A_{SM}^{'}}&{}{SM} \end{array}} \right) \end{aligned}$$

### Learning neighbor topology enhanced bi-directional random walk

The transition probabilities between slave nodes are uniformly distributed in ordinary random walks. In our bi-directional random walk, the transition probability matrix $W_{DM}^{'}$ from disease to microbial node and $W_{MD}^{'}$ from microbial network to disease network are redefined. $W_{DM}^{'}$ and $W_{MD}^{'}$ are as follows:

18 $$\begin{aligned}&W_{DM}^{'}(i,j) = \varphi \frac{{{a_{ij}}A_{SM}^{'}(i,j)}}{{\sum \nolimits _{l = 1}^{{n_m}} {{a_{il}}A_{SM}^{'}(i,l)} }} \end{aligned}$$

19 $$\begin{aligned}&W_{MD}^{'}(i,j) = \varphi \frac{{{a_{ij}}A_{SD}^{'}(i,j)}}{{\sum \nolimits _{l = 1}^{{n_d}} {{a_{li}}A_{SD}^{'}(l,j)} }} \end{aligned}$$

where $\varphi \in (0,1)$ is the jumping probability of walkers between disease network and microbial network. ${W_d}$ is the transition probability matrix between disease networks, ${W_d}(i,j)$ represents the jump probability from disease *d*(*i*) to disease *d*(*j*). ${W_d}(i,j)$ is expressed as follows:

20 $$\begin{aligned} {W_d}(i,j) = \left\{ \begin{array}{l} {{(1 - \varphi )SD(i,j)} / {\sum \nolimits _{k = 1}^{{n_d}} {SD(i,k)} }}\mathrm{{ ~~~if~ }}\sum \nolimits _{k = 1}^{{n_m}} {{a_{ik}} \ne 0} \\ {{SD(i,j)} / {\sum \nolimits _{k = 1}^{{n_d}} {SD(i,j)} }} \end{array} \right. \end{aligned}$$

Similarly, the microbial network transition probability matrix ${W_m}$ is expressed as follows:

21 $$\begin{aligned} {W_m}(i,j) = \left\{ \begin{array}{l} {{(1 - \varphi )SM(i,j)} / {\sum \nolimits _{k = 1}^{{n_m}} {SM(i,k)} }}\mathrm{{ ~~~if~ }}\sum \nolimits _{k = 1}^{{n_d}} {{a_{ki}} \ne 0} \\ {{SM(i,j)} / {\sum \nolimits _{k = 1}^{{n_m}} {SM(i,k)} }}\mathrm{{ ~~~~~~~~~~~~~otherwise}} \end{array} \right. \end{aligned}$$

In general random walk, the microbial disease transfer probability matrix and the disease microbial transfer probability matrix are transposed. Since our heterogeneous network is bi-directional, we propose an enhanced random walk to improve the comprehensive generalization ability of the bi-directional network, which can be described as follows:

22 $$\begin{aligned} P(t + 1) = (1 - r) \times W \times P(t) + r \times P(0) \end{aligned}$$

where *W* is the transition probability matrix of all nodes in the bi-directional heterogeneous network, which is defined as follows:

23 $$\begin{aligned} W = \left( {\begin{array}{*{20}{c}} {{W_d}}&{}{~~W_{MD}^{'}}\\ {~~W_{DM}^{'}}&{}{{W_m}} \end{array}} \right) \end{aligned}$$

**Fig. 2:**
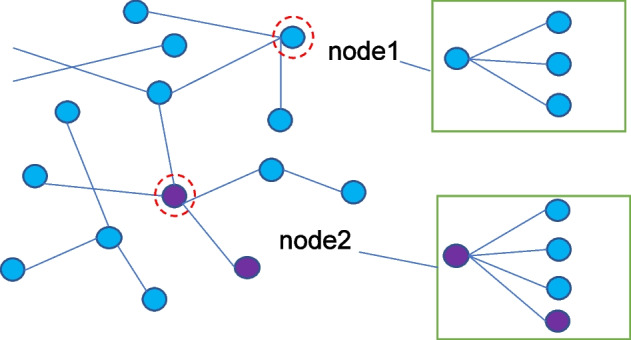
Heterogeneous map

### Selection algorithm module based on graph convolution

Most methods will aggregate all the neighbor information of nodes in heterogeneous graphs (as shown in Fig. 2), which may lead to the aggregation of redundant information to ignore the difference in local information of nodes [24]. Therefore, we adopt a graph convolution-based selection algorithm to selectively aggregate the neighbor information of each node in the network, so that our model can be applied to more practical scenarios.

**Fig. 3:**
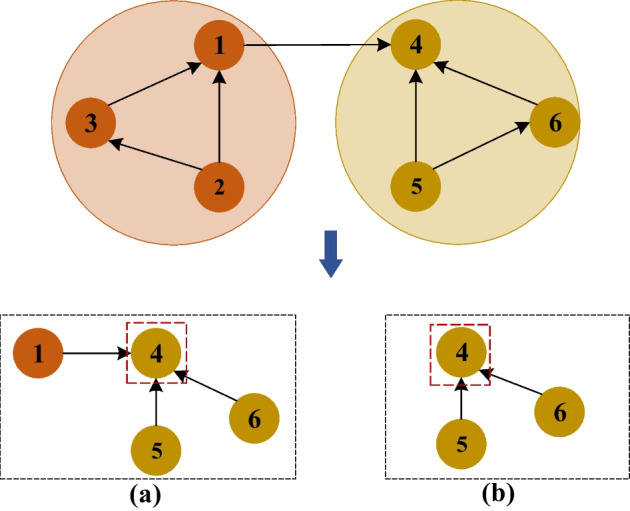
Select algorithm graph

We take the transition probability matrix *W* as the attribute matrix *X* of microbial and disease nodes, and propose a graph convolution based selection algorithm to selectively aggregate the neighbor topology and attributes of each node in the network, that is, based on graph convolution, we can aggregate them in the following two ways:

Method 1: When the graph convolution aggregates neighbors, all the neighbor information of the node is aggregated, as shown in Fig. 3a.

The adjacency matrix ${A_{all}}$ and attribute matrix *X* of the bi-directional heterogeneous network are used as the inputs of the graph convolution based selection algorithm module, and the output of the graph convolution is,

24 $$\begin{aligned} {\hat{H}} = f(x,N(x)|{\hat{\theta }} ) \end{aligned}$$

where ${\hat{H}}$ is the node feature of the convolution output of the original graph, *x* is the attribute vector of the target node, and *N*(*x*) is the attribute vector of the neighbor node of the target node.

We use the node attribute vector of graph convolution output to calculate the association probability ${\hat{y}}$ between microorganism and disease, as shown in the following formula:

25 $$\begin{aligned}&y(m(i),d(j)) = \frac{{\sum \limits _{k = 1}^m {Sim({h_{m(i)}},{h_{m(k)}})} A(j,k) + \sum \limits _{k = 1}^d {Sim({h_{d(j)}},{h_{d(k)}})} A(k,j)}}{{\sum \limits _{k = 1}^m {Sim({h_{m(i)}},{h_{m(k)}})} + \sum \limits _{k = 1}^m {Sim({h_{d(i)}},{h_{d(k)}})} }} \end{aligned}$$

26 $$\begin{aligned}&Sim(u,v) = \frac{{\sum \limits _{k = 1}^g {{u_k}{v_k}} }}{{\sqrt{\sum \limits _{k = 1}^g {u_k^2} } \sqrt{\sum \limits _{k = 1}^g {v_k^2} } }} \end{aligned}$$

where ${h_{m(i)}}$ and ${h_{d(j)}}$ represent the attribute vectors of disease node *d*(*j*) and microorganism node *m*(*i*) respectively, and *A* is the microorganism disease correlation matrix.

Method 2: In the application of graph convolution, only nodes of the same type are aggregated, as shown in Fig. 3b,

27 $$\begin{aligned} {{\hat{H}}^s} = f(x,\phi |{{\hat{\theta }} ^s}) \end{aligned}$$

where ${{\hat{H}}^s}$ is the node attribute of convolution output of graph after intervention. *x* is the node characteristic, ${\phi }$ is the causal intervention. Similarly, the microbial disease prediction association probability ${{\hat{y}}^s}$ after the intervention is also calculated based on formulas 24 and 25.

### Select algorithm

Two methods of aggregating neighbor information were used to obtain two prediction scores of microbial disease association, i.e. association probability ${\hat{y}}$ of original prediction and association probability ${{\hat{y}}^s}$ of post intervention prediction. Our purpose is that the model can make the best choice between ${\hat{y}}$ and ${{\hat{y}}^s}$ for the final prediction, so as to reduce the impact caused by the local structural difference of nodes [25], which is calculated as follows:

28 $$\begin{aligned} {\bar{y}} = h({\hat{y}},{{\hat{y}}^s}|e) \end{aligned}$$

where *h* is the selection function and *e* is the causal effect factor, which is defined as follows:

29 $$\begin{aligned} \begin{array}{l} e = f(x,N(x)|{\hat{\theta }} ) - f(x,do(N = \phi )|{\hat{\theta }} )\\ = f(x,N(x)|{\hat{\theta }} ) - f(x,\phi |{\hat{\theta }} )\\ = {\hat{y}} - {{{\hat{y}}}^s} \end{array} \end{aligned}$$

The final microbial disease association prediction probability is calculated as follows:

30 $$\begin{aligned} {\bar{y}} = \left\{ \begin{array}{l} {\hat{y}},e > = m\\ {{{\hat{y}}}^s},e < = m \end{array} \right. \end{aligned}$$

where *m* is the threshold.

## Experimental evaluation and discussion

### Parameter setting and evaluation index

Our model BDHNS runs on a GPU(Nvidia GeForceRTX2060). We quantitatively analyze the parameter random walk step number *t*, restart probability *r* and characteristic dimension *d* to determine their values. We set the restart probability *r* of random walk from 0.1 to 0.9. The embedding dimension *d* and the number of random walk steps *t* are set similarly to *r*, with *d* and *t* varying from 8 to 128 and from 5 to 30, respectively. In order to facilitate parameter tuning, one parameter is tested and the other parameters are fixed. When the restart probability *r* is 0.1, the number of steps *t* is 20, and the embedding dimension *d* is 64, our model has the best performance. We use all combinations of parameter *d*, restart probability *r* and the range of embedded dimension *d* to construct our model.

Loocv cross validation and 5-fold cross validation were used to evaluate the performance of our method and other state-of-the-art microbial disease prediction methods. In Loocv, each known association between microorganism and disease is selected as the test sample, while other known associations are training samples. In the 5-fold cross validation, the known association is regarded as a positive sample, and the unobserved association is regarded as a negative sample. All positive samples were randomly divided into five groups, four of which were put into the training set, and the rest were used for testing. In each cross validation, we randomly selected negative samples with the same amount as 4 groups of positive samples for training, and the remaining negative samples are used for testing.

Our evaluation indicators include true positive rate (TPR), false positive rate (FPR), receiver operating characteristic (ROC), and area under curve (AUC). The ROC curve can be drawn and the area under the ROC curve (AUC) can be obtained by sorting the samples with the scores of our method and different thresholds.

### Ablation experiment

Under the Five-fold cross-validation and Loocv cross-validation, the ablation experiment was used to verify the contribution of the enhanced random walk module and the graph convolution-based selection algorithm module to the prediction of microbial-disease association (Table 1). In the absence of validation of enhanced random walk, AUC decreased by 6.2% and 2.8% respectively compared with our final model. The main reason is that the enhanced random walk module enhances the neighbor topology representation of microorganisms and disease nodes, which may improve the prediction performance. Compared with the model without the selection module based on graph convolution, the AUC performance of our method is improved by 9.1% and 5.5% under five-fold cross-validation and Loocv cross-validation, respectively. This indicates that it is necessary to selectively aggregate the attributes of neighbor nodes.

**Table 1:** Results of ablation experiments on our method

Enhanced bi-directional random walk	Selection algorithm module based on graph convolution	Five fold cross validation Average AUC	Loocv cross validation Average AUC
$\times$	$\checkmark$	0.883	0.923
$\checkmark$	$\times$	0.854	0.896
$\checkmark$	$\checkmark$	0.945	0.951

### Compare other methods

**Fig. 4:**
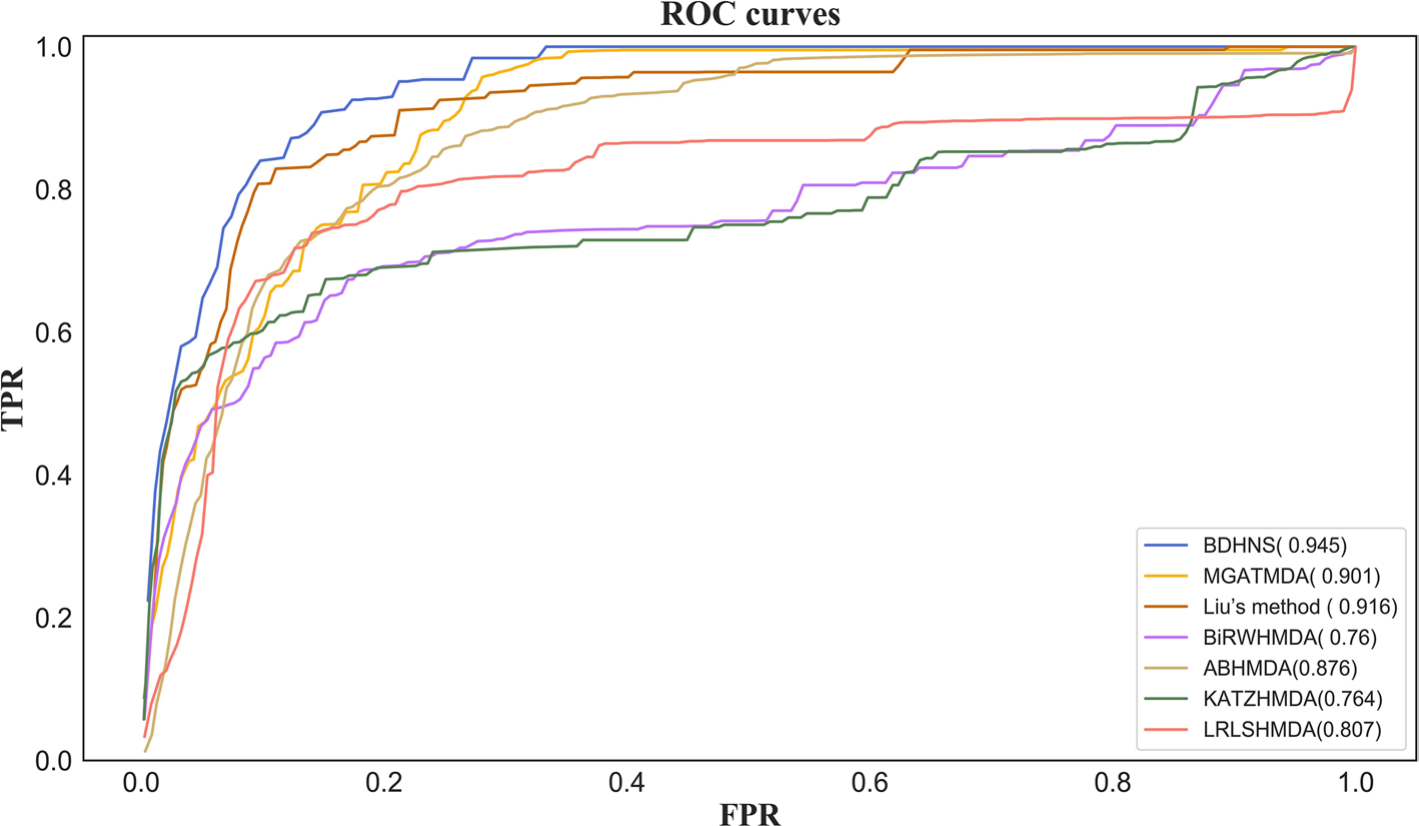
ROC curve of five methods in 5-fold cross validation

**Fig. 5:**
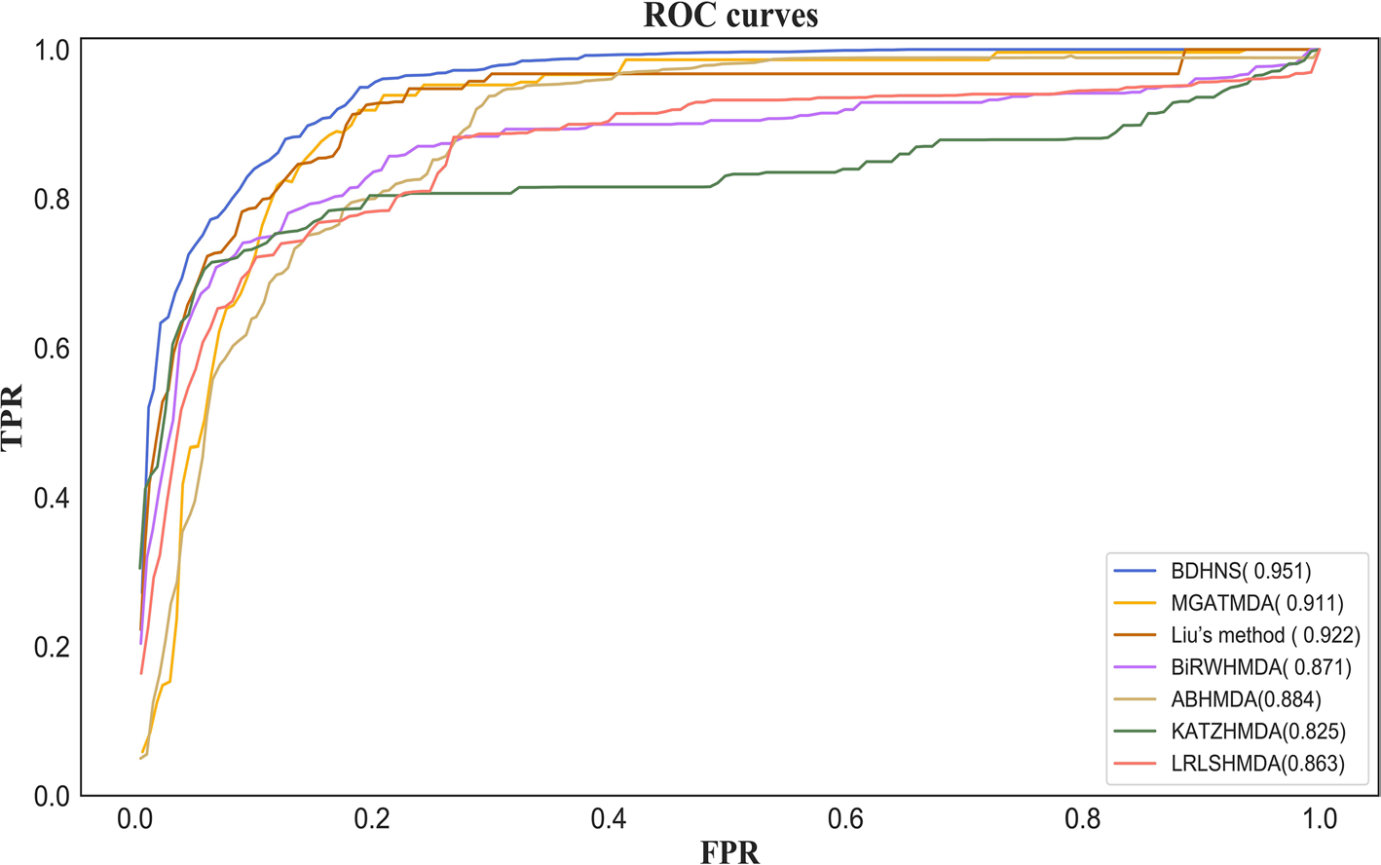
ROC curve of five methods in Loocv

We compared our method with four microbial disease association prediction methods, including BiRHMDA [4], KATZ [3], LRLSNMDA [7], ABHMDA [8], Liu’s method [18], MGATMDA [19]. In five-fold cross-validation and leave-one-out cross-validation, all methods use the same data set for training and testing. Under the five fold cross validation and Loocv cross validation, the average curves of all methods are shown in Figs. 4 and 5, respectively.

Under the five-fold cross validation, the AUC value of our model is the best, which is 0.945, which is 2.9% higher than Liu’s method, 4.4% higher than MGATMDA, 18.5% higher than BiRWHMDA method, 6.9% higher than ABHMDA method, 18.1% higher than KATZ method, and 13.8% higher than LRLSNMDA method. One possible reason why Liu’s method ranks second is that it integrates the linear features of microorganisms and diseases obtained based on non negative matrix decomposition and random walk and the nonlinear features obtained based on capsule neural network. The third ranked mgatmda utilizes the graph attention network (GAT) to deeply mine the complex association between microorganisms and diseases. BiRWHMDA, ABHMDA, LRLSNMDA and KATZ are shallow prediction methods, which are difficult to deeply integrate the multi-level attributes of microbials and diseases nodes. One possible reason why ABHMDA performs better than BiRWHMDA, LRLSNMDA and KATZ is that it integrates similarity information across multiple microorganisms. The results show that this information is helpful to predict the microbial-disease association.

Under the Loocv cross validation, the AUC value of our method is still higher than that of other methods. The AUC value of our method is 0.951, which is 2.9% , 4%, 8%, 6.7%, 12.6% and 8.8% higher than Liu’s method, MGATMDA, BiRWHMDA, ABHMDA, KATZ and LRLSNMDA, respectively.

### Case studies

In order to further evaluate the predictive performance of our method for microbial-disease association, we conducted case studies on colon cancer and ulcerative colitis. First, we can obtain the association probability of each microbial candidate disease and rank it in descending order. Then, the first 10 candidate microorganisms of each disease were selected for validation and analysis.

**Table 2:** Prediction results of top-10 Colon cancer-associated microbes

Disease name	Rank	Microbe name	Evidence
	1	Clostridia	PMID: 24603888
	2	Enterobacteriaceae	PMID: 26143056
	3	Clostridium coccoides	PMID:21850056
Colon cancer	4	Haemophilus	Unconfirmed
	5	Clostridium	PMID: 30857430
	6	Proteobacteria	PMID: 34650531
	7	Firmicutes	PMID: 34551683
	8	Bacteroidetes	PMID: 34551683
	9	Lactobacillus	PMID: 19647100
	10	Staphylococcus	PMID: 17530358

**Table 3:** Prediction results of top-10 Ulcerative colitis-associated microbes

Disease name	Rank	Microbe name	Evidence
	1	Prevotella	PMID: 16585651
	2	Clostridium difficile	PMID: 21272802
	3	Staphylococcus aureus	PMID: 21683308
Ulterative colitis	4	Haemophilus	PMID: 33748490
	5	Proteobacteria	PMID: 250187840
	6	Helicobacter pylori	PMID: 30430119
	7	Firmicutes	PMID: 30239655
	8	Erysipelotrichaceae	PMID: 32169445
	9	Coriobacteriaceae	PMID: 31812509
	10	Clostridia	Unconfirmed

Colon cancer is a life-threatening malignant tumor. The risk of colon cancer is closely related to intestinal flora [25]. In this study, 9 of the top 10 candidate microorganisms related to colon cancer predicted by our method have been confirmed by experiments, as shown in Table 2. Clostridia may promote Colon cancer [26]. It is found that enterobacteriaceae and proteobacteria are very common in colon cancer [27, 28]. Sidhu et al. [29] was found that the bacteria that can produce butyrate in the intestinal microbiota of colon cancer patients were greatly reduced. Clostridium coccoides is one of the bacteria that can produce butyrate. There is evidence that clostridium is associated with colon cancer [30]. Compared with normal people, fermicutes and bacteroidetes in colon cancer patients decreased and increased significantly respectively [31]. Lactobacillus has immunoregulatory effect on human colon cancer cells [32]. Studies have proved that staphylococcus can prevent cancer [33].

Ulterative colitis is a chronic inflammatory disease of colon and rectum. Its pathogenesis is closely related to intestinal microbial imbalance [34]. Methods 9 of the top 10 candidate microorganisms related to ulcerative colitis predicted by us have been confirmed by experiments, as shown in Table 3. There is evidence that prevotella, clostridium difficile and proteobacteria have a great impact on the pathogenesis of ulcerative colitis [35–37]. Gryaznova et al. [38] reported patients with ulcerative colitis caused by staphylococcus aureus. There is evidence that haemophilus is increased in patients with ulcerative colitis [39]. Helicobacter pylori is also involved in the pathogenesis of ulcerative colitis [40]. The abundance of firmicutes in lymph nodes of patients with ulgenerative colitis is very high [41]. Erysipelotrichaceae was found to be a key bacterium related to ulcerative colitis [42]. It was found that the number of coriobacteriaceae decreased in patients with ulcerative colitis [43].

In conclusion, case studies of two diseases show that our method can indeed find potential microbial disease associations.

## Summary

We propose a new microbial disease prediction method to learn and integrate multiple characteristics of diseases and microorganisms. Bi-directional heterogeneous networks are established to help integrate similarities and associations between microorganisms and diseases. An enhanced random walk module is established to learn the neighbor topology information of microorganisms and disease nodes. In order to selectively aggregate node features, a graph convolution-based selection algorithm is further established. In the Loocv and 5-fold cross-validation, the improved performance of our method in AUC was demonstrated by comparing with several microbial disease prediction models. The performance of our method is further demonstrated by the case studies of colon cancer and ulcerative colitis. In the future, we can integrate more types of data, such as gene sequencing and human metabolite data, to help predict the potential association between microorganism and disease. In addition, nodes in heterogeneous networks can be connected through different semantic meta paths. Therefore, in the future, we will integrate the information from the meta path in our method.

## Data Availability

The datasets analyzed during the current study are downloaded from the website http://www.cuilab.cn/hmdad. The datasets used during the current study available from the corresponding author on reasonable request.
